# Inhaled pH-Responsive polymyxin B-loaded albumin nanoparticles against pneumonia caused by carbapenem resistant *Klebsiella pneumoniae*

**DOI:** 10.1016/j.mtbio.2025.101590

**Published:** 2025-02-18

**Authors:** Ziling Li, Huiling Lei, Jiannan Hu, Tong Zhou, Shuaiqi Yuan, Xinyue Ma, Yunfei Zhu, Chao Liu, Decai Wang, Yuzhou Wu, Shuyun Xu

**Affiliations:** a Department of Respiratory and Critical Care Medicine, Tongji Hospital, Tongji Medical College, Huazhong University of Science and Technology, Wuhan, 430030, Hubei Province, China; b Key Laboratory of Respiratory Diseases of National Health Commission, Tongji Hospital, Tongji Medical College, Huazhong University of Sciences and Technology, Wuhan, 430030, Hubei Province, China; c Hubei Key Laboratory of Bioinorganic Chemistry and Materia Medica, Hubei Engineering Research Center for Biomaterials and Medical Protective Materials, School of Chemistry and Chemical Engineering, Huazhong University of Science and Technology, Wuhan, 430074, Hubei Province, China

**Keywords:** *Klebsiella pneumoniae*, Carbapenem resistance, Pneumonia, Nanoparticles, Pulmonary delivery

## Abstract

The pneumonia induced by carbapenem resistant *Klebsiella pneumoniae* (CRKP) has high morbidity and mortality. Among the antibiotics currently available, polymyxin B (PMB) is considered to be the last line of defense. Routine intravenous administration of PMB has many problems, such as severe neurotoxicity and nephrotoxicity. In this study, a novel inhaled PMB-loaded albumin nanoparticles (PEG-pHSA@PMB) capable of penetrating airway mucus and responding to the infection microenvironment is constructed. An acid-responsive functional molecule (PEBA) and NH_2_-PEG-SH are linked to the surface of human serum albumin (HSA) via the conjugation reaction. Subsequently, PMB is loaded through electrostatic interactions to yield PEG-pHSA@PMB. The sulfhydryl groups of PEG-pHSA@PMB interact with mucins to help penetrate mucus after inhaled. In an acidic environment, the protonation of the tertiary amino groups within PEG-pHSA@PMB causes the charge alteration, which leads to the release of PMB. It demonstrated excellent mucus permeability, potent bactericidal activity, and superior bacteriostatic effects compared to sole PMB. Inhalation of PEG-pHSA@PMB significantly reduced the bacterial load in the lungs of mice with CRKP pneumonia, alleviating inflammatory response. Moreover, PEG-pHSA@PMB exhibited good cytocompatibility and biosafety. The novel strategy of the inhalation drug delivery system is promising for the treatment of pneumonia caused by drug-resistant bacteria.

## 1 Introduction

Pneumonia is a significant disease that poses a threat to human health. Over 2.49 million people die from pneumonia annually, surpassing the number of deaths caused by tuberculosis (1.18 million deaths) and HIV infection (864 000 deaths) [1]. *Klebsiella pneumoniae* (Kp) is the main bacterium identified in respiratory specimens [2]. With the extensive application of antibiotics, the progress of antibiotic resistance is growing increasingly severe, particularly the carbapenem resistant organism (CRO) that causes hospital-acquired pneumonia and ventilator-associated pneumonia (VAP). The detection rate of carbapenem resistant *Klebsiella pneumoniae* (CRKP) has been rising year after year [3]. The pooled mortality due to CRKP infections is between 33 % and 50 % [4]. At present, the effective antibiotics against CRKP are highly limited, and it is exceedingly difficult to treat CRKP pneumonia.

Polymyxin is deemed as the last line of defense against refractory CRO [5]. Polymyxin binds to the lipopolysaccharide and phospholipids of the outer membrane of gram-negative bacteria through electrostatic action, and destroys the stability of the cell membrane, ultimately causing the bacteria to perish [6]. There exist several issues in the treatment of CRKP pneumonia with polymyxin, such as the narrow therapeutic window, poor pulmonary permeability, and severe systemic adverse reactions. Compared to intravenous administration, the inhalation route can enhance the cumulative concentration and retention time of drugs in the lungs, maximizing the efficacy while reducing the risk of drug exposure in other organs in order to lessen systemic side effects [7]. The results from a multicenter cohort study indicate that additional polymyxin B (PMB) inhalation can improve clinical outcomes in patients with VAP caused by extensively drug-resistant Kp [8]. Another study found that inhalation of PMB is superior to polymyxin E (PME, also known as colistin) in terms of antimicrobial efficacy, but PMB inhalation is associated with a higher incidence of bronchospasm than PME [9]. Inhalation of PMB alone is difficult to penetrate the airway mucus barrier and reach the distal lung tissue. PMB is cytotoxic to lung epithelial cells, and direct inhalation can damage lung tissue [10].

Nano-drug delivery system can enhance the availability of drugs and reduce toxicity, possessing great development potential. Innovative nano-antibiotics can offer a potent means for systemic or local drug-resistant bacterial infections [11]. Chai et al. prepared a PMB-polysaccharide polyion nanocomplex via the electrostatic interaction between positively charged PMB and negatively charged 2,3-dimethyl maleic anhydride (DA) grafted chitoligosaccharide (CS), CS-DA/PMB [12]. It can be disassembled to release PMB in an acidic environment. Intratracheal injection of CS-DA/PMB was effective and safe in the treatment of acute *Pseudomonas aeruginosa* pneumonia in mice. In another study, a polymer nanocarrier for sustained PMB release was prepared to treat mice with multidrug-resistant *Acinetobacter baumannii* pneumonia via aerosol inhalation [13]. Wu et al. devised a mucus-permeable nanoparticle (HA@PLGA-PMB) for aerosol inhalation to deliver PMB by employing hydrophilic hyaluronic acid (HA) [14]. HA was combined with a water-in-oil system containing a poly (lactic-co-glycolic acid) (PLGA) copolymer of PMB. The PMB delivery systems mentioned above have failed to accommodate both nebulized inhalation and intelligent drug release, nor has it been applied in a mouse model of CRKP pneumonia. We propose to construct a novel type of PMB delivery system to penetrate the airway mucus, reach the distal lung tissue, and release PMB intelligently at the site of infection to reduce systemic adverse reactions.

Human serum albumin (HSA) has many advantages as a common drug delivery vector. HSA is a negatively charged protein with a molecular weight (MW) of 66.5 KDa. The positively charged PMB can be electrochemically bound to the sites situated between the HSA domains I and III within the body [15]. HSA exhibits good biocompatibility, stability and degradability. One study also found that the drug delivery systems combined with HSA might potentially enhance lung permeability and drug absorption following inhalation administration [16]. The HSA-based drug delivery systems have been applied to treat sepsis-related acute lung injury, acute respiratory distress syndrome, pulmonary fibrosis, pulmonary tuberculosis, and lung squamous cell carcinoma [[17], [18], [19], [20], [21], [22]]. No drug delivery system based on the HSA vector has been used to treat pneumonia induced by bacterial infection so far.

When acute lung infection occurs, the airway goblet cells secrete additional mucins, which together with water, carbohydrate, protein, lipid, and mineral secreted by glands form airway mucus [23]. The thickened mucus layer will undoubtedly prevent the nano-drug delivery system from entering the tissue. The mucin monomers in the mucus are mostly cross-linked by disulfide bonds. N-acetylcysteine is a commonly employed aerosolized expectorant in the clinic. It contains sulfhydryl groups that can be exchanged with the disulfide bonds to disintegrate the mucins and DNA fibers, decreasing the viscosity and elasticity of the sputum [24,25]. The surface modification of sulfhydryl groups on the nanoscale drug delivery systems may be an effective strategy to help penetrate the airway mucus. Polyethylene glycol (PEG) is a classic viscous inert material, which aids in increasing the hydrophilicity of the surface of nano-delivery system [26,27]. Due to its low toxicity and good biocompatibility, PEG is a polymer approved by the U. S. Food and Drug Administration and has been widely used in biomedicine [28].

Microenvironment-responsive drug delivery systems can achieve targeted drug delivery, decrease drug side effects, and improve drug efficacy. The stimulus-responsive polymers with dynamic properties have attracted considerable attention. Their structures or properties can alter in response to internal or external stimulators [29,30]. The pH value is the most representative internal stimulation in physiological environments such as pathological parts and acidic organelles [31,32]. The airway exchange was impaired at the infection site, and CRKP underwent glycolysis under hypoxia, and the acid products were increased. The pH value of the infected area decreased to approximately 5.0–6.5, which was lower than that of the normal lung tissue and the airway surface lining fluid of about 7.0 under the physiological condition [11,33]. The two main mechanisms for drug release from pH-sensitive nanomedicine delivery systems are pH-sensitive chemical bond breaking and protonation of chemical groups [34]. By utilizing bromelain as a carrier, the researchers developed a novel pH-sensitive nanocarrier through ortho-transesterification [35]. As the transesterification bond was broken, the release of the drug increased as the pH value decreased. On the other hand, nano-materials frequently comprise pH-responsive chemical groups such as histidine, polyhistidine, tertiary amine, and sulfonamide. Under acidic conditions, the protonation of tertiary amine groups can trigger the transition from hydrophobicity to hydrophilicity of the nanomedicine delivery system, resulting in the breakdown of the nanomedicine to release the encapsulated drug [36,37].

**Scheme 1:**
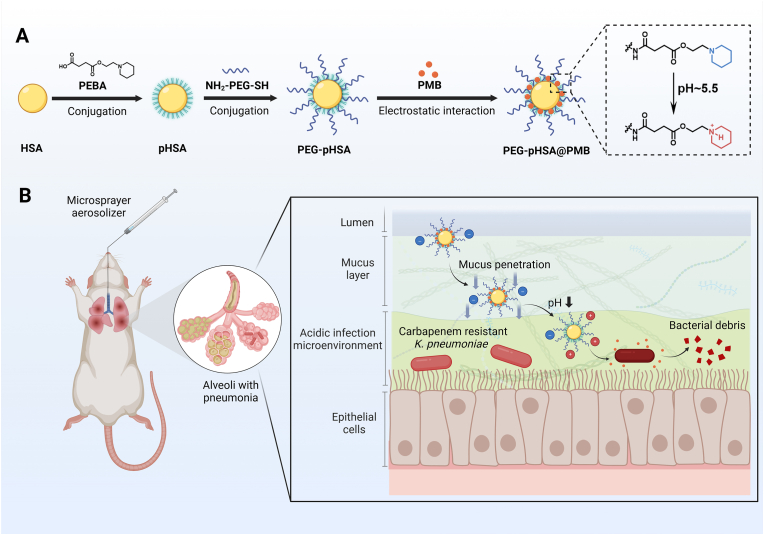
The schematic illustration of the preparation and in vivo antibacterial activity of PEG-pHSA@PMB nanoparticles. HSA, human serum albumin; PEBA, 4-oxo-4-(2-(piperidin-1-yl)ethoxy)butanoic acid; PMB, polymyxin B. Created by BioRender.com.

In this study, novel pH-responsive PMB-loaded HSA nanoparticles were constructed upon an innovative strategy of airway mucus penetration as well as an acid microenvironment response strategy. Scheme 1 presents the hypothesis of the study. We modified the pH-response molecule and amine-PEG-thiol in turn on HSA, and loaded the PMB by electrostatic interaction. Due to the consumption of amino groups in the synthesis process, the carrier is negatively charged, which could provide anionic components during self-assembly. PMB could be used as a cationic component electrostatic self-assembly. In vitro and in vivo experiments were conducted to explore the antibacterial activity and safety of nanoparticles. The novelty of our work lies in the construction of a novel PMB delivery system and its successful application in inhalation administration. Our study provides a theoretical basis for future clinical application and a new therapeutic strategy for the treatment of drug-resistant bacterial pneumonia.

## 2 Materials and methods

### 2.1 Materials

Albumin from human serum (≥96 %) and gelatin were purchased from Sigma-Aldrich (Missouri, USA). Polymyxin B sulfate was obtained from MedChemExpress (New Jersey, USA). 1-Ethyl-3(3-dimethyl aminopropyl) carbodiimide (EDC, 99 %) was purchased from Energy Chemical (Shanghai, China). N-hydroxysuccinimide (NHS, 99 %) was purchased from Civi Chemical Technology (Shanghai, China). Succinic anhydride (98 %) and sulfo-cyanine5 succinimidyl ester were provided by Macklin Biochemical Technology (Shanghai, China). 2-Piperidinoethanol and trifluoroacetic acid was purchased from Senrise Technologies (Anhui, China). Acetonitrile (HPLC grade, ≥99.9 %), low molecular-weight salmon sperm DNA, and diethylene triamine pentaacetic acid (≥98 %) was purchased from Aladdin Biochemical Technology (Shanghai, China). Amine-PEG2000-Thiol (NH_2_-PEG-SH) and Methoxy-PEG2000-Amine (mPEG-NH_2_) were provided by Ponsure Biotech (Shanghai, China). Coomassie brilliant blue was brought from Sangon Biotech (Shanghai, China). LB broth, MacConkey inositol adonitol carbenicillin (MIAC) agar, and egg yolk emulsion was brought from Hope Bio-Technology (Qingdao, China). Agar was purchased from Mym Biological Technology (Kuala Lumpur, Malaysia). Mucin from pig stomach mucosa was brought from Yuanye Bio-Technology (Shanghai, China). Acetone, potassium hydroxid, potassium chloride, and sodium chloride was obtained from Sinopharm Chemical Reagent (Shanghai, China). The Roswell Park Memorial Institute (RPMI)-1640 medium was obtained from KeyGen Biotech (Jiangsu, China). Cell counting kit-8 was brought from Yeasen Biotechnology (Shanghai, China). Deionized water (18.2 MΩ cm) was generated by a Heal Force ultrapure water system (Shanghai, China).

### 2.2 Synthesis of 4-oxo-4-(2-(piperidin-1-yl)ethoxy)butanoic acid (PEBA)

0.3 g succinic anhydride (3 mmol, 1 eq) was dissolved in 3 ml of acetonitrile and 0.3873 g of N-hydroxyethyl piperidine (3 mmol, 1 eq) was added dropwise under nitrogen atmosphere, the mixture was refluxed at 80 °C for 6 h and purified by the flash-column chromatography to get PEBA as an oily substance (0.60 g, 88 % yield). (^1^H NMR (400 MHz, CDCl_3_) δ 9.17 (s, 1H), 4.31 (t, *J* = 5.5 Hz, 2H), 2.92 (t, *J* = 5.5 Hz, 2H), 2.80 (s, 4H), 2.55 (dd, *J* = 5.7, 3.6 Hz, 4H), 1.77–1.67 (m, 4H), 1.51 (d, *J* = 4.4 Hz, 2H).

### 2.3 Preparation of PEG-pHSA@PMB and mPEG-pHSA@PMB

4 mg of HSA were dissolved in 200 μL of phosphate buffered saline (PBS) buffer with a pH of 7.4 to prepare a 20 mg/mL HSA solution for use. 12 mg of PEBA were dissolved in 200 μL of PBS, followed by the addition of 22.8 mg of EDC and 13.8 mg of NHS, and then oscillated at 800 rpm, 25 °C for 1 h. The solution was then added to the HSA solution and magnetically stirred in a 25 °C water bath for 18 h. The reaction solution was transferred to Vivaspin 500 centrifuge tubes with a molecular weight cutoff (MWCO) 30 KDa and centrifuged at 4 °C, 8000 rpm for 10 min. The pH-responsive HSA (pHSA) solution was obtained by washing with deionized water several times. 8 mg of EDC and 4 mg of NHS were added to the pHSA solution. After being activated at room temperature for 1 h, 8 mg of NH_2_-PEG-SH (MW 2 KDa) at the HSA: NH_2_-PEG-SH molar ratio of 1:67 were added to the solution. Under the condition of a 25 °C water bath, the solution reacted for 12 h with magnetic agitation in the dark. It was centrifuged again, and the washing solution was collected as described previously, then the PEG-pHSA solution was obtained. Finally, 3 mg of PMB were dissolved in 100 μL of deionized water. The PMB solution was added to the PEG-pHSA solution drop by drop, then mixed on a thermal mixer at 25 °C and 800 rpm for 12 h. The PEG-pHSA@PMB nanoparticle solution was obtained by centrifuging with Vivaspin 500 (MWCO 30 KDa) at 4 °C and 8000 rpm for 10 min. After being freeze-dried with a vacuum freeze-dryer, the solid was stored in a −20 °C refrigerator. We used mPEG-NH_2_ (MW 2 KDa) instead of NH_2_-PEG-SH (MW 2 KDa) to synthesize nanoparticles without sulfhydryl groups, mPEG-pHSA@PMB, and the rest of the steps were the same as above.

### 2.4 Characterizations

The ^1^H NMR spectrum of PEBA was recorded on a Bruker AV400 spectrometer (Rheinfelden, Switzerland). The mass spectrum of PEBA was determined by a Thermo Scientific UltiMate 3000-microTOFII high performance liquid chromatography - time of flight mass spectrometry (Massachusetts, America). The morphology and size of nanoparticles were observed through a HT7700 transmission electron microscopy (TEM) (Hitachi, Japan). The zeta potential of nanoparticles was measured by a Zetasizer Nano ZS dynamic light scattering (DLS) equipment (Malvern, England).

### 2.5 Calculation of encapsulation efficiency (EE) and loading capacity (LC)

The standard PMB solutions with the concentrations of 0.1, 0.3, 0.5, 0.7, 1.0, 1.5, 2.5 mg/mL were prepared using deionized water. Chromatograms of standard PMB were obtained via a LC100 high-performance liquid chromatography (HPLC) instrument (Shanghai, China). The HPLC chromatographic conditions were as follows, chromatographic column: SHODEX chromatographic column C18-100-5 4E (4.6 mm × 250 mm, 5 μm); mobile phase: 0.5 ‰ trifluoroacetic acid solution for pump A, and acetonitrile solution containing 0.5 ‰ trifluoroacetic acid for pump B; gradient elution procedure: pump A was kept at 100 % within 0–1 min, pump B was kept at 5 % within 1–10 min, and pump B was kept from 5 % to 20 % within 10–40 min. The detection wavelength was 215 nm, the column temperature was 25 °C, the flow rate was 1.0 mL/min, and the sample volume was 20 μL. Taking the concentration of PMB as the horizontal coordinate and the area of HPLC peak as the vertical coordinate, the standard curve of PMB was drawn and the equation was obtained.

The flow through from the ultrafiltration after the addition of PMB in the preparation of nanoparticles was collected for HPLC analysis to measure the concentration of free PMB. The EE and LC of PEG-pHSA@PMB nanoparticles were calculated according to the following formulas.

$$EE\left(\%\right)=\left(1-\frac{\left[encapsulant\;in\;permeate\;solution\right]}{\left[encapsulant\;added\right]}\right)\times 100\%$$

$$LC\left(\%\right)=\frac{\left[weight\;of\;loaded\;drug\right]}{\left[weight\;of\;drug\;infeed\right]}\times 100\%$$

### 2.6 Drug release test

The PEG-pHSA@PMB solution with a mass concentration equivalent to 4 mg/mL of loaded PMB was prepared using deionized water. Double volumes of PBS (pH 7.4 or pH 5.5) were added respectively, and the mixture was oscillated at 37 °C. After reacting for 1, 2, 4, 8, 12, 24, 48 h separately, the solution was placed into Vivaspin 500 (MWCO 30 KDa). Following centrifugation at 4 °C and 5000 rpm for 30 min, the concentration of PMB in the flow through was determined by HPLC analysis. The drug release efficiency (E_r_) of PMB at different times was obtained. We calculated the E_r_ of PEG-pHSA@PMB at different points in time by employing the following formula.

$$E_{r}\left(\%\right)=\frac{\left[volume\;of\;waste\right]\times \left[concentration\;of\;PMB\;in\;the\;waste\right]}{\left[weight\;of\;loaded\;drug\right]}\times 100\%$$

### 2.7 Mucus penetration experiment in vitro

10 % (w/v) gelatin solution was prepared with deionized water by means of melting in the oven. 1 mL of liquid gelatin was added to each glass vial, and then placed at room temperature until it was set. The artificial mucus was prepared in vitro according to the formula in the reference, encompassing 250 mg of salmon sperm DNA, 125 mg of mucin derived from pig stomach mucosa, 0.1475 g of diethylene triamine pentaacetic acid (DTPA), 125 mg of NaCl, 55 mg of KCl, 125 μL of 50 % egg yolk emulsion, 500 μL of RPMI-1640 medium, and 25 mL of deionized water [14]. The pH of artificial mucus is about 4.0–5.0. 2 mL of artificial mucus was added to the surface of the gelatine in each vial. PEG-pHSA@PMB solution (at a concentration equivalent to 4 mg/mL of loaded PMB) was prepared. 500 μL of PEG-pHSA@PMB was mixed with 500 μL of Coomassie brilliant blue solution (1 g/L), and then incubated on a shaker at room temperature for 2 h. 100 μL of the successfully stained PEG-pHSA@PMB was added to every surface of artificial mucus drop by drop. The vials were incubated in a 37 °C incubator, and photographed at 0, 0.5, 1, and 2 h to observe the color of mucus penetration. Two hours later, the vials were taken out from the incubator and cooled to room temperature. When the gelatin solidified, we poured out the top layer of artificial mucus. The gelatin layer was washed several times with deionized water, and then melted in a 37 °C incubator for 30 min to make the gelatin layer liquefied. After blowing and mixing, the absorbance of the gelatin layer at 595 nm was measured using a light absorption microplate reader. The experiment was repeated three times.

### 2.8 Determination of the minimal inhibitory concentration (MIC) and minimum bactericidal concentration (MBC) of nanoparticles

The CRKP (strain number: DSM30104) and carbapenem-resistant *Acinetobacter baumannii* (CRAB, strain number: DAM30007) utilized in our study was isolated from respiratory secretions of patients suffering from pneumonia in the respiratory intensive care unit of Tongji Hospital affiliated to the Huazhong University of Science and Technology. The suspension of CRKP at a concentration of 3 × 10^6^ colony forming units (CFU)/mL was prepared using sterile PBS. According to the guideline of the American Clinical and Laboratory Standards Institude, both broth dilution and standard plate counting were employed for the susceptibility testing of PMB, PEG-pHSA@PMB, and PEG-pHSA in pH 5.5. The inoculated 96-well plates were incubated in a 37 °C incubator for 18 h. The absorption value at 600 nm (OD_600_) was measured by means of a microplate reader. The MIC value was the lowest concentration proximate to the OD_600_ value of the negative control wells, while the MBC value was the lowest concentration of an antibacterial agent that brings about a 99.9 % reduction of the initial bacterial inoculum. An additional 100 μL of culture solution was drawn from the wells with low OD_600_ values in the aforementioned 96-well plates to the LB agar plates. Plate colony counts were performed after 24 h of incubation at 37 °C to determine the MBC value.

### 2.9 In vitro antibacterial effect

Firstly, the initial concentration of 1 × 10^6^ CFU/mL was obtained by the re-suspension of CRKP with sterile PBS solution (pH 5.5) in the static germicidal efficacy test. The concentrations of PMB were 4, 8, 16 μg/mL in the 5 mL suspension of CRKP with different drugs. The CRKP content was gauged by the standard plate after being incubated for 4 h in a constant temperature oscillator at 37 °C and 180 rpm. A 1 × 10^8^ CFU/mL of CRAB suspension was treated with 4 μg/mL PEG-pHSA@PMB (equivalent to loaded PMB) for 3 h, and then the amount of CRAB was counted via the standard plate.

In the test of antibacterial kinetics, the relationship between the OD_600_ value measured by turbidimetric method and the number of viable bacteria counted by the plate counting method was determined. Subsequently, 4, 8, and 16 μg/mL PMB, PEG-pHSA@PMB (corresponding to the concentration of loaded PMB), and PEG-pHSA (the concentration of the carrier after removing the loaded PMB) were separately added into the 10 mL bacterial solution with an OD_600_ of 0.4. The pH of the solution was regulated to 5.5. Under the conditions of 37 °C and 140 rpm, the OD_600_ value of the bacterial solution was determined at 0, 1, 2, 3, 4, 6, 8, 10, 12, 15, 18, 21, and 24 h respectively to sketch the bacteriostasis curves. Each experiment was replicated three times.

### 2.10 Cytotoxicity test

Human bronchial epithelial (HBE) cells were sourced from the American model culture collection. HBE cells were uniformly spread in 96-well plates at a density of 4000 cells per well on the day prior to drug treatment. Once the HBE cells had grown overnight and adhered to the wall, the experimental groups were substituted with complete medium containing different concentrations of PMB, PEG-pHSA, and PEG-pHSA@PMB. The control groups were supplanted with a fresh, drug-free, and complete medium. After the cells were cultivated in a 37 °C incubator for 24 h, the old medium in each well was drawn clean, and the fresh complete medium containing 10 % (v/v) cell counting kit-8 (CCK-8) was added to each well in a dark setting. The cells were cultivated at 37 °C for 3 h. The absorbency of the pores at 450 nm was measured, and the cell viability was calculated according to the absorbency.

### 2.11 In vivo antibacterial experiment

The ICR male mice, aged 6–8 weeks and weighing 30–34 g, were selected. The mice were purchased from Beijing Vitonlihua Experimental Animal Technology Co., Ltd, with the license number of SCXK (Hubei) 2022-0030. A total of 160 mice were used in this study. All the mice were kept in separate cages in a Specific Pathogen Free laboratory with a temperature ranging from 18 °C to 25 °C and a humidity ranging from 50 % to 70 %. The experimental mice used in this study had been ethically certified by the welfare ethics review board of Tongji hospital, and the animal ethics batch number was TJH-202203004.

We constructed the models of CRKP pneumonia in mice according to the method described in Ref. [38]. The mice were anesthetized by the intraperitoneal injection of 1 % sodium pentobarbital solution. A total of 30 μL CRKP suspension (1 × 10^8^ CFU/mL) was inoculated into the airway of the mice using a BioJane microsprayer aerosolizer (Shanghai, China). At the second hour after the mice were infected with CRKP, the first treatment was administered through the microsprayer aerosolizer in the lungs of the mice, and the second treatment was given at the fourth hour in scheme A (Fig. 3F). The scheme B was to treat the mice at 12 h and 18 h post-infection, respectively (Fig. 3F). 30 μL PEG-pHSA@PMB solution (equivalent to 4 mg/kg loaded PMB), PMB (4 mg/kg), or PEG-pHSA solution (the concentration of the carrier after removing the loaded PMB) were administered each time. The mice in the control group were treated with 30 μL sterile PBS solution (pH 7.4).

After 24 h, the mice were euthanized, and bronchoalveolar lavage fluid (BALF) was collected to count inflammatory cells, including neutrophils and macrophages. A moderate amount of right upper lung tissue was cut for homogenization. After continuous dilution of 100 μL solution, it was spread on MIAC medium and cultured in a 37 °C incubator. The load of CRKP was then counted after 18 h. The right lower lungs of the mice were taken for real-time quantitative reverse transcription polymerase chain reaction (RT-qPCR) to detect the inflammatory factors. The primer sequences used in this study were as follows: mice-derived glyceraldehyde-3-phosphate dehydrogenase (GAPDH), forward: TGGCCTTCCGTGTTCCTAC, reverse: GAGTTGCTGTTGAAGTCGCA; mice-derived tumor necrosis factor-α (TNF-α), forward: CAGGCGGTGCCTATGTCTC, reverse: CGATCACCCCGAAGTTCAGTAG; mice-derived interleukin-6 (IL-6), forward: TAGTCCTTCCTACCCCAATTTCC, reverse: TTGGTCCTTAGCCACTCCTTC; mice-derived Chemokine (C-X-C motif) ligand 1 (CXCL1), forward: TGTGGGAGGCTGTGTTTGTA, reverse: ACGAGACCAGGAGAAACAGG. The left lung tissues of mice were collected for hematoxylin-eosin (HE) staining. The HE sections of lung tissue of the mice were observed under a light microscope with a magnification of 200 times. The severity of lung injury in each group was scored according to the Mikawa method in reference [39]. The following four targets were graded from 0 to 4 points: alveolar congestion; pulmonary hemorrhage; neutrophils infiltration, and septal thickening. 0 = the lightest injury, 1 = the mild injury, 2 = the moderate injury, 3 = the severe injury, and 4 = the most severe injury. The lung injury score was the summary of the four variables.

### 2.12 Distribution of PEG-pHSA@PMB in lungs

We used sulfo-cyanine5 succinimidyl ester to label PEG-pHSA and then loaded PMB to obtain Cy5-labeled PEG-pHSA@PMB. At the second hour after the mice were infected with CRKP (1 × 10^8^ CFU/mL), 30 μL Cy5-labeled PEG-pHSA@PMB, Cy5-labeled PEG-pHSA solution, or PBS were administered using a BioJane microsprayer aerosolizer. After 24 h, the mice were euthanized, and the left lung tissues of mice were collected for frozen sections. Diamidino-phenyl-indole (DAPI) nuclear staining was used. The sections of lung tissue of the mice were observed under a fluorescence microscope with a magnification of 200 times.

### 2.13 In vivo safety evaluation

Healthy male ICR mice of equal weight were selected. PBS, sole PMB (8 mg/kg), PEG-pHSA@PMB (equivalent to 8 mg/kg loaded PMB), and PEG-pHSA solution (the nanoparticle mass after removing the loaded PMB) were administered by nebulizer inhalation in 30 μL three times a day. The survival of mice was observed after 3 days of nebulized inhalation, and samples were taken 4 h after the last dose. The lungs, kidneys, brains, hearts, livers and spleens of mice in each group were collected and immersed in 4 % paraformaldehyde solution. With the same experimental procedure as the HE staining of mouse lung tissue, the organs were stained with HE. The histological changes were observed through a microscope.

### 2.14 Data processing

GraphPad Prism 9.0 (GraphPad software, USA) was used to analyze the data. The normally distributed data were presented as mean ± standard deviation (SD). Two-tailed Student's *t*-test was used for the comparison between two groups. One-way analysis of variance was applied for the comparison among multiple groups. Bonferroni's method was used for post hoc testing. P < 0.05 was considered statistically significant.

## 3 Results and discussion

### 3.1 Preparation and characterization of PEG-pHSA@PMB

Firstly, the structure of resulting PEBA was confirmed by ^1^H NMR (Fig. S1A) and mass spectrum (Fig. S1B). In the acronym for nanoparticle (PEG-pHSA@PMB), we used “p” to refer to its pH-responsiveness. The final PEG-pHSA@PMB nanoparticles presented a spherical morphology with a diameter of 320–400 nm, as detected by TEM (Fig. 1A). The zeta potentials of PEG-pHSA@PMB at pH 5.5 and pH 7.4 were measured by DLS (Fig. 1B). It was observed that the zeta potential of PEG-pHSA@PMB nanoparticles changed from −5.44 mV (at pH 7.4) to −0.51 mV (at pH 5.5), as the tertiary amine groups within the nanoparticles were protonated in the acidic environment. This result preliminarily indicated that the nanoparticles were responsive to pH, which might facilitate drug release.

**Fig. 1:**
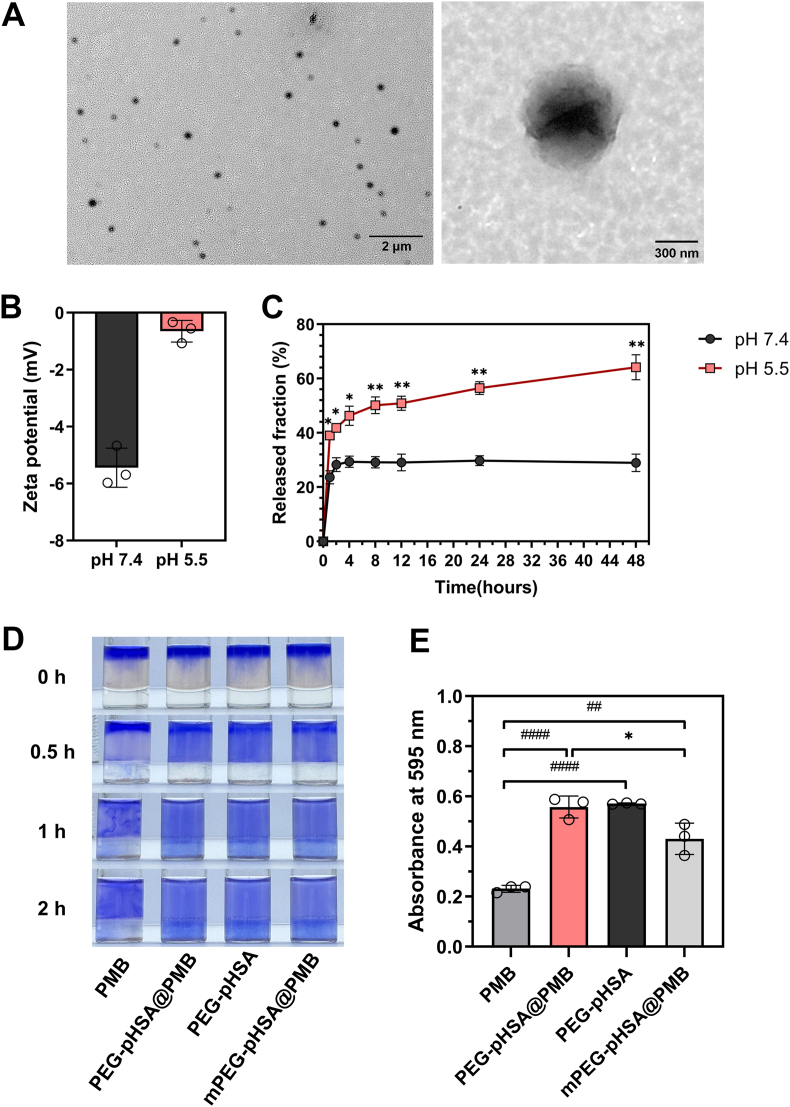
Characterization of PEG-pHSA@PMB nanoparticles. (A) TEM of PEG-pHSA@PMB. PEG-pHSA@PMB was spherical with a diameter ranging from 320 to 400 nm. The scale bar on the left 2 μm, and the scale bar on the right is 300 nm. (B) Zeta potential of PEG-pHSA@PMB nanoparticles at different pH values. The zeta potential of nanoparticles in pH 7.4 and pH 5.5 was measured via DLS, with n = 3. (C) Drug release curves of PEG-pHSA@PMB nanoparticles under different pH conditions. The E_r_ of PEG-pHSA@PMB nanoparticles at different times in pH 7.4 and pH 5.5 was measured by HPLC. Data were presented as the mean ± standard deviation, with n = 3. ∗Compared with pH 5.5, ∗P < 0.05, ∗∗P < 0.01. (D) The mucus penetration of different drugs. The penetration of blue PEG-pHSA@PMB, PMB, PEG-pHSA, and mPEG-pHSA@PMB in the artificial mucus (middle layer) was observed. The bottom layer was a colorless transparent gelatin layer, with n = 3. (E) Absorbance of the gelatin layer after penetration of different drugs into artificial mucus. The absorbency of gelatin layer (bottom layer) at 595 nm was measured using a light absorption microplate reader, with n = 3. ^#^Compared to sole PMB, ^##^P < 0.01, ^####^P < 0.0001. ∗Compared with PEG-pHSA@PMB, ∗P < 0.05.

Next, we detected the LC and EE of PEG-pHSA@PMB using HPLC. The LC was (33.55 ± 2.36) %, and EE was (53.12 ± 1.70) %. The drug release experiment revealed that the E_r_ of PEG-pHSA@PMB was (39.06 ± 1.25) % at pH 5.5 and (23.62 ± 1.95) % at pH 7.4 after 1 h (∗P < 0.05). After 48 h, the E_r_ in the acid environment was (64.19 ± 3.77) %, significantly higher than that in the neutral environment (28.94 ± 2.61) % (∗∗P < 0.01). The results indicated that PEG-pHSA@PMB possessed an excellent ability to rapidly release the drug in response to acids (Fig. 1C).

To evaluate the mucus penetration performance of PEG-pHSA@PMB, we constructed artificial mucus models in vitro by the method described by Wu et al. [14]. PEG-pHSA@PMB penetrated the mucus faster and deeper over time compared to sole PMB, suggesting that PEG-pHSA@PMB was better at penetrating mucus in vitro than PMB alone (Fig. 1D). As shown in Fig. 1E, the absorbance of the gelatin layer at 595 nm in the PEG-pHSA@PMB group or PEG-pHSA group was significantly higher than that in the sole PMB group, further verifying the mucus penetration ability of PEG-pHSA@PMB (^####^P < 0.0001). In order to verify the role of sulfhydryl groups in mucus penetration, we used mPEG-NH_2_ (MW 2 KDa) instead of NH_2_-PEG-SH (MW 2 KDa) to synthesize mPEG-pHSA@PMB. The absorbance of the gelatin layer in the mPEG-pHSA@PMB group was significantly lower than that in the PEG-pHSA@PMB group (∗P < 0.05) in Fig. 1E. The results suggest that sulfhydryl groups in the PEG-pHSA@PMB nanoparticles could help penetrate mucus more efficiently. The particle-mediated effects and PEG may also be involved in mucus penetration, as the mPEG-pHSA@PMB penetrated the artificial mucus more efficiently than PMB (^##^P < 0.01). There may be a number of factors that contribute to the superior mucus penetration ability of PEG-pHSA@PMB.

The ideal mucus-penetrating nano-delivery system has the characteristics of small particle size, non-electropositive surface and good hydrophilicity [40]. It has been reported that large-sized (>250 nm) nanoparticles can penetrate the mucus layer more effectively [41]. The PEG-pHSA@PMB nanoparticles were negatively charged under neutral conditions, thus they did not interact with negatively charged mucins and were not trapped in the mucus. The PEG modified on the surface of PEG-pHSA@PMB nanoparticles could increase hydrophilicity. PEG-pHSA@PMB meets the basic requirements of a pulmonary nano-drug delivery system with an appropriate size, potential, and hydrophilicity to overcome the mucus barrier for inhaled drugs. Furthermore, the sulfhydryl groups on the surface of the PEG-pHSA@PMB nanoparticles can break the disulfide bonds and reduce cross-linking of mucin fibers. The results of the artificial mucus penetration in vitro indicated that the PEG-pHSA@PMB nanoparticles had favorable properties of mucus penetration.

The ability of nanoparticles to respond to the microenvironment plays a role in the local drug release after penetrating the mucus. The pH-sensitive molecule in our study had a tertiary amino group that could be protonated in the acidic environment. The results of the zeta potential and drug release curve in different pH demonstrated that the PEG-pHSA@PMB nanoparticles possessed good pH-responsive properties.

### 3.2 In vitro antibacterial activity of PEG-pHSA@PMB

We determined the MIC and MBC against the CRKP strain to evaluate the bactericidal activity of PEG-pHSA@PMB at pH 5.5 (as presented in Table 1). The MIC of PEG-pHSA@PMB was 8 μg/mL, while the MBC was 32 μg/mL, suggesting that the antimicrobial activity of the nanoparticles primarily originated from the release of PMB in the acidic environment. Combined with the LC (33.55 % approximately) and E_r_ (64.19 % in pH 5.5 approximately) of PEG-pHSA@PMB, we estimated that the concentration of PMB released by 8 μg/mL PEG-pHSA@PMB nanoparticles at pH 5.5 is about 1.72 μg/mL, which was close to the MIC (2 μg/mL) of PMB. The difference in MIC and MBC values between PEG-pHSA@PMB and PMB was reasonable. In the meantime, the MIC and MBC of PEG-pHSA were >1024 μg/mL, indicating that the carriers without PMB had no substantial effect on bacteriostasis or bactericidal efficacy.

**Table 1:** The MIC and MBC of different drugs in the acidic environment.

	MIC (μg/mL)	MBC (μg/mL)
PMB	2	16
PEG-pHSA@PMB	8	32
PEG-pHSA	>1024	>1024

The bactericidal activity of PEG-pHSA@PMB in vitro was detected via the standard plates counting assay (Fig. 2A). PEG-pHSA@PMB exhibited the same bactericidal activity as PMB alone when being incubated with 1 × 10^6^ CFU/mL CRKP for 4 h. The result once again indicated that the antimicrobial activity of the nanoparticles was attributed to the efficient release of PMB in the acidic environment. Subsequently, we conducted a bacteriostasis experiment to further evaluate the bacteriostatic effect of PEG-pHSA@PMB in vitro. The relationship between the OD_600_ measured by turbidimetry and colony counts was as follows: Y = 3539796748 ∗ X - 582809959, and R^2^ = 0.9715, where X was OD_600_ and Y was CFU/mL (Fig. 2B). Different sample solutions with concentrations of 4, 8, and 16 μg/mL (equivalent to loaded PMB) were incubated with the CRKP suspension at pH 5.5. The CRKP solutions were incubated at 37 °C for 24 h, and the OD_600_ values were measured at different time points to plot the bacterial growth curve. After 10 h of incubation, PEG-pHSA@PMB demonstrated a significantly stronger bacteriostatic effect compared to the control group (∗∗∗P < 0.001), while there was no difference between the PMB group and the PBS group (Fig. 2C).

**Fig. 2:**
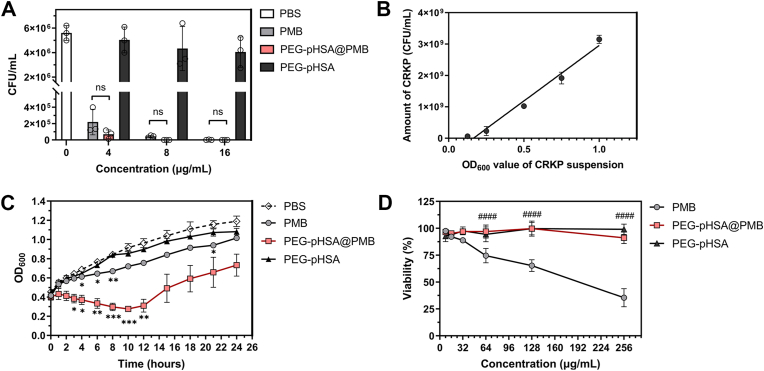
The outcomes of antibacterial experiments with CRKP in vitro. (A) The static germicidal effect of PEG-pHSA@PMB nanoparticles. A 1 × 10^6^ CFU/mL of CRKP suspension was treated with different concentrations of drugs for 4 h, and then the amount of CRKP was counted via the standard plate, n = 3. (B) The linear relationship between OD_600_ and the number of CRKP. The correlation between the OD_600_ measured by turbidimetry and the colony count measured by standard plate counting was as follows: Y = 3539796748 ∗ X - 582809959, R^2^ = 0.9715, where X was OD_600_ and Y was CFU/mL, n = 3. (C) The bacteriostasis curves of the drugs. The OD_600_ value of the CRKP suspension was determined at different times, n = 3. ∗Compared with the PBS group, ∗P < 0.05, ∗∗P < 0.01, ∗∗∗P < 0.001. (D) The cytotoxicity assessment of PEG-pHSA@PMB nanoparticles. The toxicity of PMB, PEG-pHSA@PMB, and PEG-pHSA to HBE cells was detected by the CCK-8 kit, n = 4. ^#^Compared to sole PMB, ^####^P < 0.0001.

**Fig. 3:**
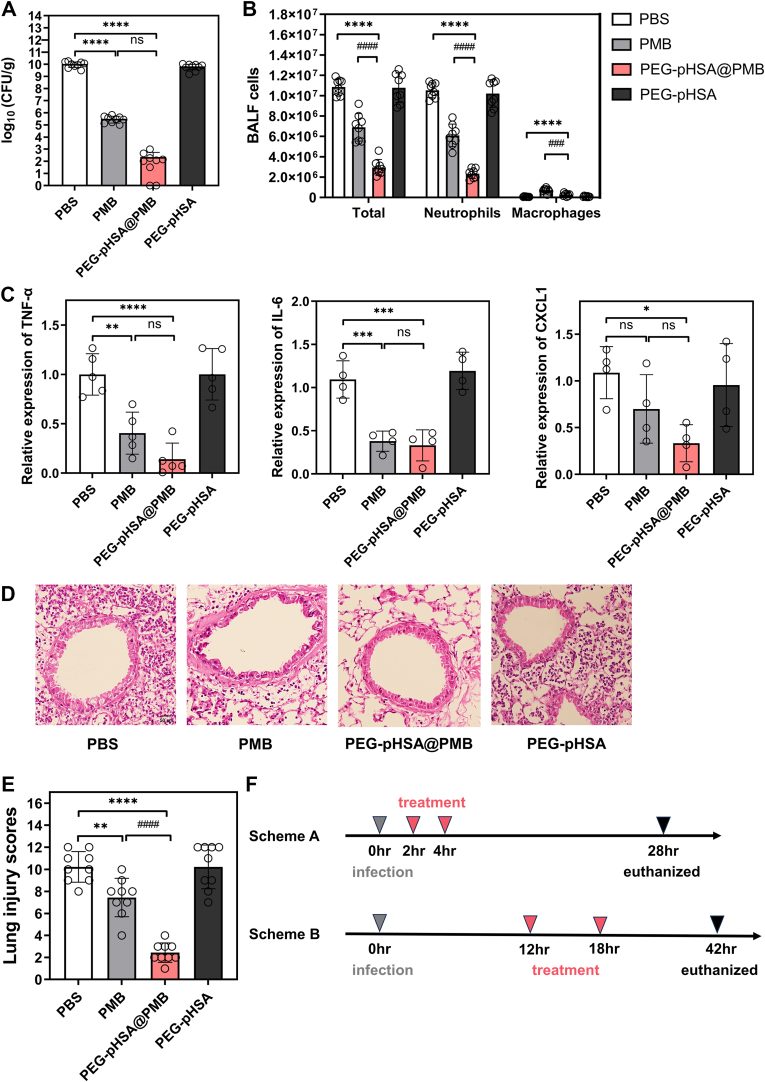
The therapeutic effect and safety of PEG-pHSA@PMB nanoparticles on acute CRKP pneumonia in mice. (A) The load of CRKP in the lungs of mice that inhaled different drugs. The bacterial load of CRKP in the lungs of pneumonia mice treated with inhaled drugs (equivalent to 4 mg/kg loaded PMB) was measured by the bacterial plate counting method, n = 9. ∗Compared with the PBS group, ∗∗∗∗P < 0.0001. (B) The number of total cells, neutrophil cells, and macrophages in the BALF of mice (n = 8). ∗Compared with the PBS group, ∗∗∗∗P < 0.0001. ^#^Compared with sole PMB, ^###^P < 0.001, ^####^P < 0.0001. (C) The levels of TNF-α, IL-6, and CXCL1 in the lungs of mice treated with different drugs. The levels of TNF-α, IL-6, and CXCL1 mRNA in the right lower lung of mice were detected by RT-qPCR, n = 4 or 5. ∗Compared with the PBS group, ∗P < 0.05, ∗∗P < 0.01, ∗∗∗P < 0.001, ∗∗∗∗P < 0.0001. (D) HE staining of lung sections of CRKP pneumonia mice treated with different drugs. The scale bar is 50 μm. (E) The statistical chart of lung injury score by HE staining of lung tissue in each group, n = 9. ∗Compared with the PBS group, ∗∗P < 0.01, ∗∗∗∗P < 0.0001. ^#^Compared with sole PMB, ^####^P < 0.0001. (F) Schematic of experimental design for treatment timescales of post-infection with CRKP.

Carbapenem-resistant *Acinetobacter baumannii* (CRAB) is another common strain that causes the death of pneumonia patients clinically [42,43]. Similarly, PEG-pHSA@PMB showed comparable bactericidal activity with the PMB group in vitro against CRAB in Fig. S2A. Then 4 μg/mL PEG-pHSA@PMB (equivalent to loaded PMB) were incubated with CRAB suspension at pH 5.5. After 3 h of incubation, PEG-pHSA@PMB demonstrated a significantly stronger bacteriostatic effect compared to the PMB group (^##^P < 0.01), and the bacteriostatic effect of the PEG-pHSA@PMB was also significantly better than that of PMB with P < 0.05 at the first and second hour after incubation (Fig. S2C). It can be concluded that the PEG-pHSA@PMB is more effective than PMB in inhibiting both CRKP and CRAB for a certain period of time. We speculated that it may be related to the sudden release of large amounts of PMB from nanoparticles over a period of time.

### 3.3 Bronchial epithelial cell cytotoxicity

PMB has lung epithelial cytotoxicity, while the toxicity of PMB-loaded nanoparticles remains unclear. The cytotoxicity of the samples was examined through incubation with HBE cells. A typical dose-dependent toxicity was observed in the cells treated with PMB alone (Fig. 2D). The viability of HBE cells gradually decreased along with the increasing dose of PMB. When the concentration of PMB ranged from 64 μg/mL to 256 μg/mL, it presented obvious toxicity to HBE cells. The cell viability dropped to 36 % when the concentration of PMB was 256 μg/mL. On the contrary, more than 90 % of the cells survived after being treated with PEG-pHSA@PMB within a wide range of 8–256 μg/mL (equivalent to loaded PMB), suggesting that PEG-pHSA@PMB could be used in vivo without causing cytotoxicity.

### 3.4 CRKP pneumonia mice treated with PEG-pHSA@PMB

We further explored the antibacterial efficacy of PEG-pHSA@PMB via the treatment of CRKP pneumonia mice. The CRKP pneumonia mice presented with the following symptoms: sluggishness, decreased appetite, rough hair, a tendency to close their eyes, shortness of breath, and cyanosis of the ear and tail. The mice were treated with aerosol inhalation of the drugs respectively at the second and fourth hour after being infected with CRKP in scheme A (Fig. 3F). To evaluate the in vivo efficacy of the nanoparticles, we harvested and homogenized the lungs after 24 h, and then counted the number of CRKP within them via the standard plate counting method (Fig. 3A). The number of CRKP in the lungs of the PBS group was approximately 1.05 × 10^10^ CFU/g. After being treated with PEG-pHSA@PMB (equivalent to 4 mg/kg loaded PMB), the number of CRKP in the lungs decreased to 2.24 × 10^2^ CFU/g, which was significantly lower than that of the PBS group (∗∗∗∗P < 0.0001). The lungs treated with PMB alone still retained 3.27 × 10^5^ CFU/g of CRKP, which was higher than that in the PEG-pHSA@PMB group, and there was no significant difference between the two groups. By counting the number of cells in the BALF, we found that the inhalation of PEG-pHSA@PMB could reduce the airway neutrophil inflammation more significantly than PMB alone with P < 0.0001 (Fig. 3B). We also detected the levels of inflammatory factors in the lungs of mice treated with different drugs by RT-qPCR. As illustrated in Fig. 3C, the levels of TNF-α and IL-6 in the lungs of the mice with the treatment of PEG-pHSA@PMB was significantly lower than that of the PBS group (P < 0.0001 and P < 0.001, respectively). Compared with PBS, PEG-pHSA@PMB treatment significantly reduced the CXCL1 levels in the lungs with P < 0.05, while there was no significant difference in PMB treatment. As the most well-known acute pro-inflammatory cytokines, TNF-α and IL-6 are critical for host resistance to Kp [44]. TNF-α is an important cytokine that initiates inflammatory and bactericidal processes [45]. TNF-α levels can increase early in the infection. IL-6 is a prominent pro-inflammatory cytokine with a range of inflammatory roles [46]. CXCL1 is also necessary for Kp elimination by hosts [44,47].

The HE staining of the lungs further supported that the antibacterial activity of the nanoparticles was better than that of PMB (Fig. 3D). There were inflammatory cells infiltration around the airways and the destruction of alveolar structures in the PBS group. PEG-pHSA@PMB was more effective in alleviating the above pathological changes than PMB. The statistical results of the lung injury scores in Fig. 3E suggested that the degree of lung injury in mice treated with PEG-pHSA@PMB was milder than that of PMB (^####^P < 0.0001). We also examined the distributions of nanoparticles and carriers using Cy5-labeled PEG-pHSA@PMB and PEG-pHSA in Fig. S3. They were distributed in the alveoli, suggesting that the nanoparticles can reach distal lung tissue.

**Fig. 4:**
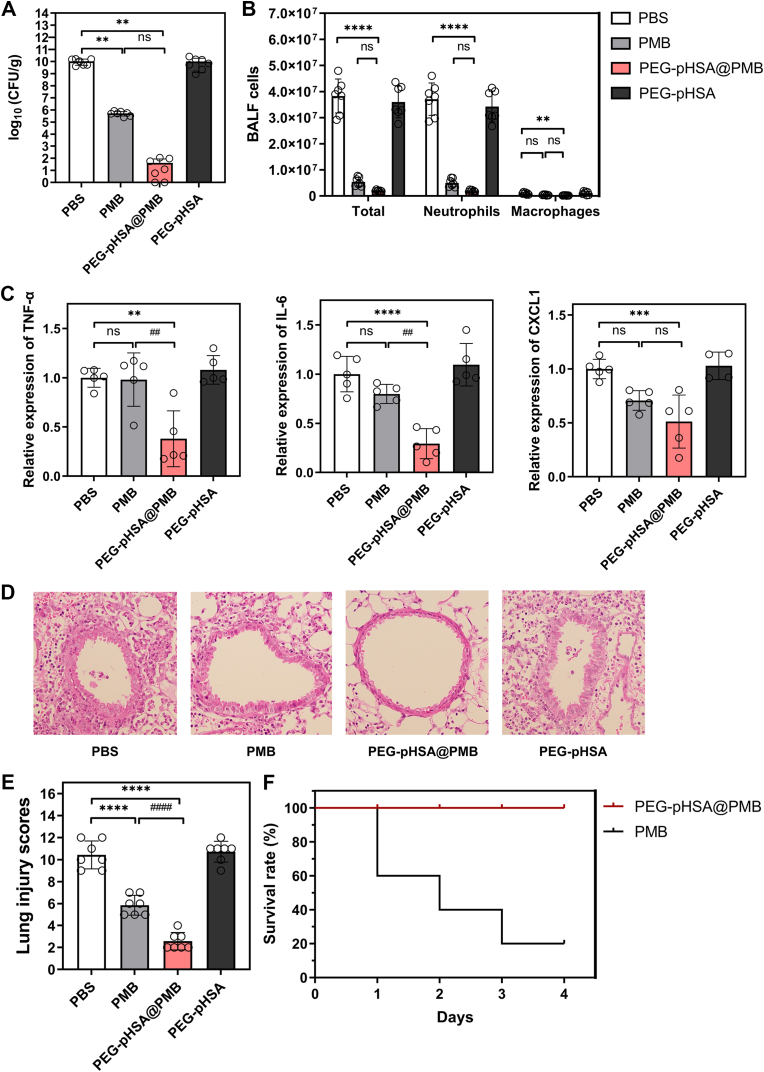
The therapeutic effect of PEG-pHSA@PMB nanoparticles on CRKP pneumonia in scheme B. (A) The load of CRKP in the lungs of mice that inhaled different drugs. The bacterial load of CRKP in the lungs of pneumonia mice treated with inhaled drugs (equivalent to 4 mg/kg loaded PMB) was measured by the bacterial plate counting method, n = 7. ∗Compared with the PBS group, ∗∗P < 0.01. (B) The number of total cells, neutrophil cells, and macrophages in the BALF of mice (n = 7). ∗Compared with the PBS group, ∗∗P < 0.01, ∗∗∗∗P < 0.0001. (C) The levels of TNF-α, IL-6, and CXCL1 in the lungs of mice treated with different drugs. The levels of TNF-α, IL-6, and CXCL1 mRNA in the right lower lung of mice were detected by RT-qPCR, n = 5. ∗Compared with the PBS group, ∗∗P < 0.01, ∗∗∗P < 0.001, ∗∗∗∗P < 0.0001. ^#^Compared with sole PMB, ^##^P < 0.01. (D) HE staining of lung sections of CRKP pneumonia mice treated with different drugs. (E) The statistical chart of lung injury score by HE staining of lung tissue in each group, n = 7. ∗Compared with the PBS group, ∗∗∗∗P < 0.0001. ^#^Compared with sole PMB, ^####^P < 0.0001. (F) The survival of mice treated with different drugs. The survival of healthy ICR mice was observed after inhaling 30 μL of different drugs (equivalent to 8 mg/kg loaded PMB) three times daily for 3 days, n = 5.

To validate the efficacy of the nanoparticles in a longer-term infection model, we employed scheme B (Fig. 3F). The number of CRKP in the lungs was approximately 1.04 × 10^10^ CFU/g in the PBS group, 5.25 × 10^5^ CFU/g in the PMB group (4 mg/kg), and 41 CFU/g in the PEG-pHSA@PMB (equivalent to 4 mg/kg loaded PMB) group (Fig. 4A). PEG-pHSA@PMB could also significantly reduce the airway neutrophil inflammation in mice with CRKP pneumonia (Fig. 4B). As illustrated in Fig. 4C, the levels of TNF-α and IL-6 in the PEG-pHSA@PMB group were significantly lower than that of the PMB group (^##^P < 0.01). Compared with PBS, only PEG-pHSA@PMB significantly reduced the CXCL1 levels in the lungs with P < 0.001 (Fig. 4C). The HE staining of the lungs showed that the antibacterial activity of the nanoparticles was also better than that of PMB in this scheme (Fig. 4D). The lung injury scores in Fig. 4E suggested that the degree of lung injury in mice treated with PEG-pHSA@PMB was milder than that of PMB (^####^P < 0.0001).

On the one hand, PEG-pHSA@PMB could be able to reach the site of infection more effectively with its good mucus permeability. On the other hand, PEG-pHSA@PMB could release PMB rapidly in the area of bacterial infection with an acidic response to the microenvironment of infection. Consequently, nebulized PEG-pHSA@PMB was more effective in vivo than PMB alone. The inhalation of PEG-pHSA@PMB decreased the bacterial load in the lungs, the inflammatory cell counts in the BALF, the inflammatory factors, and alleviated the pathological changes in the lungs.

### 3.5 Histological toxicity in vivo

**Fig. 5:**
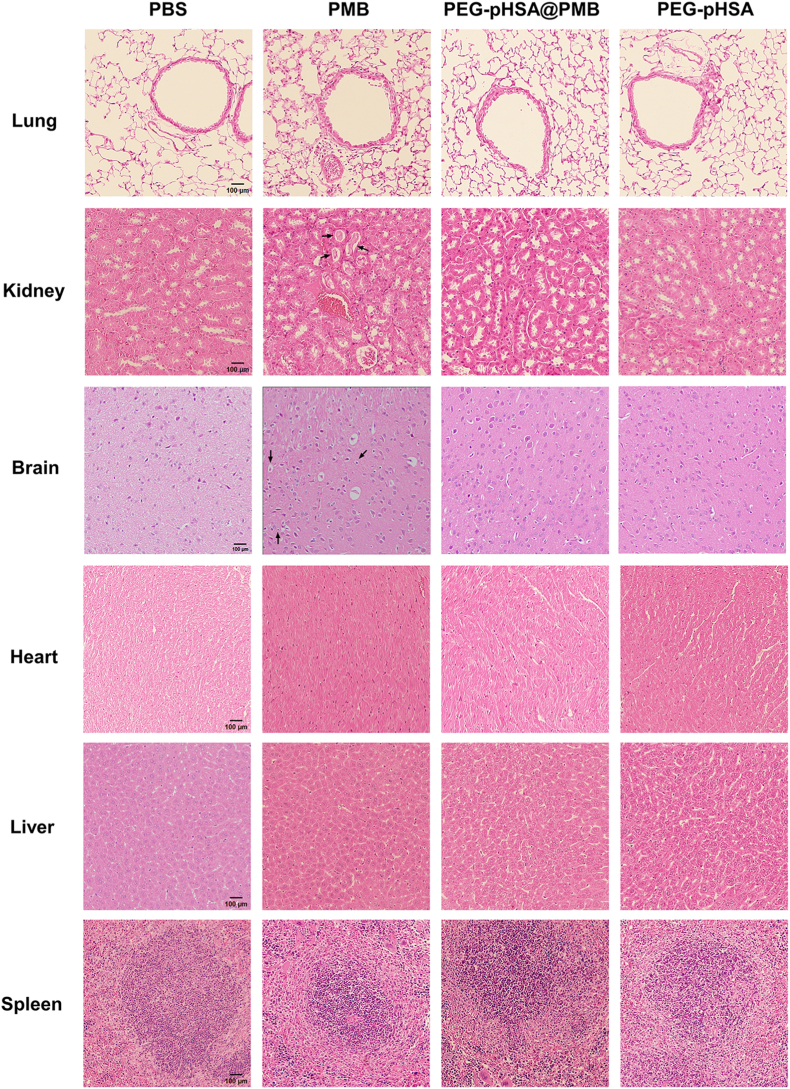
HE staining of different tissues in mice treated with different drugs. The HE staining results of the lungs, kidneys, brains, hearts, livers, and spleens of ICR mice were examined after inhaling 30 μL of different drugs (equivalent to 8 mg/kg loaded PMB) three times daily for 3 days, n = 5. The scale bar is 100 μm.

Healthy mice were nebulized with 30 μL of different solutions (equivalent to 8 mg/kg loaded PMB) by a microsprayer three times daily, which lasted for 3 days. The survival rate of mice treated with PMB was 60 % on the first day, 40 % on the second day, and 20 % on the third day (Fig. 4F). The 3-day survival rate of the other groups was 100 %. The HE staining of the lungs, kidneys, brains, hearts, livers, and spleens of mice treated with different drugs was analyzed to evaluate the safety in vivo (Fig. 5). Nephrotoxicity and neurotoxicity of PMB have already been reported, so the focus should be on the pathological changes of the kidneys and brains in addition to the lungs. Inflammatory cells infiltration in the lungs, protein deposit in the renal tubules, and atrophy of cerebral neurons were observed in mice treated with PMB alone. There were no obvious pathological changes in the lungs, kidneys, and brains of the mice treated with PEG-pHSA@PMB or PEG-pHSA. No obvious toxicity was found in the hearts, livers, and spleens of all groups.

## 4 Conclusions

The novel PMB nano-delivery system designed and synthesized in our study not only was suitable for inhalation, but also possessed good antibacterial efficacy and safety in vitro as well as in vivo. This study offered a novel and effective strategy for the inhalation drug delivery system in the treatment of pneumonia, which could potentially be a promising drug delivery system for treating pulmonary infections caused by drug-resistant bacteria. PMB was loaded through electrostatic interaction to construct the final nanoparticles, thus the system in our study might also be applied to deliver similar types of drugs to effectively treat lung diseases. However, there are some limitations in this study. We did not study the distribution of nanoparticles in the lungs in detail after nebulized inhalation, nor did we explore the pharmacokinetics and effect kinetics of nanoparticles in vivo. We also have not studied the efficacy of nanoparticles in models of pneumonia caused by bacteria other than CRKP. Further exploration will be conducted in the future.

## CRediT authorship contribution statement

**Ziling Li:** Writing – review & editing, Writing – original draft, Methodology, Investigation, Formal analysis, Data curation, Conceptualization. **Huiling Lei:** Writing – review & editing, Methodology, Investigation, Formal analysis, Conceptualization. **Jiannan Hu:** Software, Methodology, Data curation. **Tong Zhou:** Writing – review & editing, Methodology, Investigation, Formal analysis. **Shuaiqi Yuan:** Software, Methodology, Data curation. **Xinyue Ma:** Resources, Methodology. **Yunfei Zhu:** Investigation, Formal analysis. **Chao Liu:** Methodology, Formal analysis. **Decai Wang:** Software, Resources. **Yuzhou Wu:** Writing – review & editing, Methodology, Investigation, Formal analysis, Conceptualization. **Shuyun Xu:** Writing – review & editing, Supervision, Resources, Project administration, Conceptualization.

## Funding

This study was supported by the grant from 10.13039/501100001809National Natural Science Foundation of China (81370134).

## Declaration of competing interest

The authors declare that they have no known competing financial interests or personal relationships that could have appeared to influence the work reported in this paper.

## Figures and Tables

**figs1:**
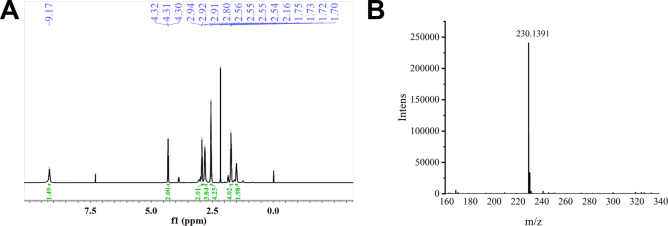
Structural characterization of PEBA. (A) 400 MHz proton NMR spectrum of PEBA in CDCl_3_. (B) Mass spectrum of PEBA.

**figs2:**
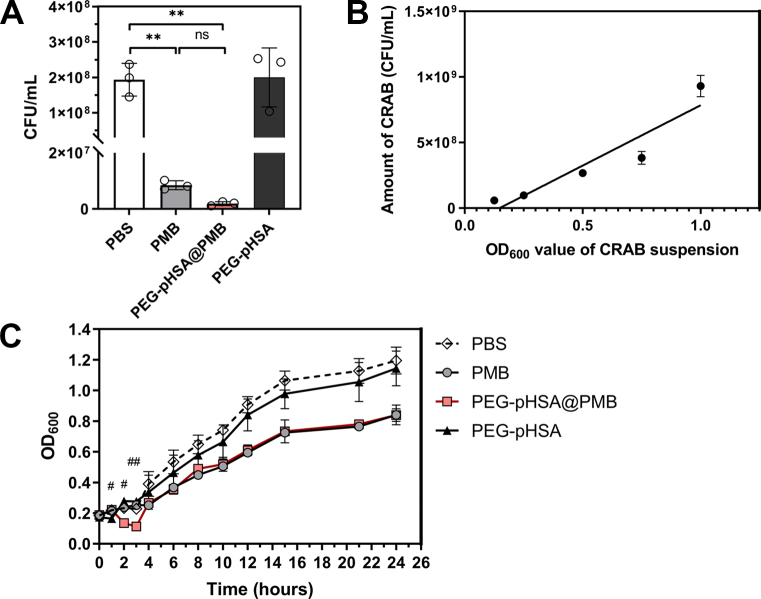
The outcomes of antibacterial experiments with CRAB in vitro. (A) The static germicidal effect of PEG-pHSA@PMB nanoparticles. A 1×10^8^ CFU/mL of CRAB suspension was treated with 4 μg/mL PEG-pHSA@PMB (equivalent to loaded PMB) for 3 hours, and then the amount of CRAB was counted via the standard plate, n = 3. (B) The linear relationship between OD_600_ and the number of CRAB. The correlation between the OD_600_ measured by turbidimetry and the colony count measured by standard plate counting was as follows: Y = 920373984∗X - 136396341, and R^2^ = 0.8664, where X was OD_600_ and Y was CFU/mL, n = 3. (C) The bacteriostasis curves of the drugs. The OD_600_ value of the CRAB suspension was determined at different times, n = 3. ∗Compared with the PBS group, ∗∗P<0.01. ^#^Compared to sole PMB, ^#^P<0.05, ^##^P<0.01.

**figs3:**
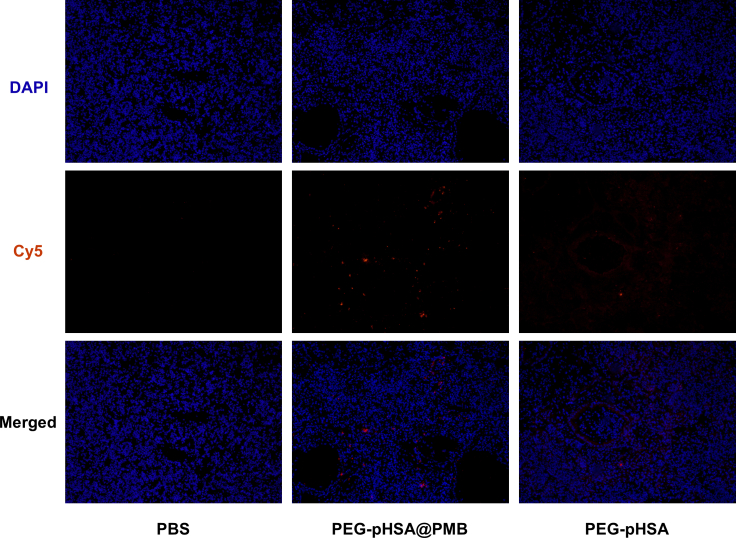
The distributions of Cy5-labeled PEG-pHSA@PMB and PEG-pHSA were observed under a fluorescence microscope with 200 magnifications. The left lung tissues of mice were collected for frozen sections after inhaling 30 μL of Cy5-labeled PEG-pHSA@PMB and PEG-pHSA, n = 3. Cy5-labeled PEG-pHSA@PMB (red fluorescence), Cy5-labeled PEG-pHSA (red fluorescence), and nuclei (DAPI, blue fluorescence).

## Data Availability

Data will be made available on request.
